# Salivary exosomal *Mycobacterium tuberculosis* DNA enables sensitive detection of paucibacillary tuberculosis: a molecular diagnosis study

**DOI:** 10.3389/fcimb.2026.1811140

**Published:** 2026-06-01

**Authors:** Jun Ma, Xubin Zheng, Yifan He, Xuewen Tang, Zhen Huang, Li Wang, Xing Tang, Yuanyuan Huang, Zhizhi Lei, Min Zhu, Wei Sha

**Affiliations:** 1 Department of Tuberculosis, Shanghai Pulmonary Hospital, School of Medicine, Tongji University, Shanghai, China; 2 Clinic and Research Center of Tuberculosis, Shanghai Pulmonary Hospital, School of Medicine, Tongji University, Shanghai, China; 3 Division of Infectious Diseases, Department of Medicine, Karolinska Institute, Stockholm, Sweden; 4 Department of Infectious Diseases, Karolinska University Hospital, Stockholm, Sweden; 5 Shanghai Liquidbio Bio-tech Co., Ltd., Shanghai, China; 6 Center for Cellular and Molecular Diagnostics, Tulane University School of Medicine, New Orleans, LA, United States; 7 Department of Medicine, Tulane University of Medicine, New Orleans, LA, United States; 8 Shanghai Key Laboratory of Tuberculosis, Shanghai Pulmonary Hospital, School of Medicine, Tongji University, Shanghai, China

**Keywords:** exosome, molecular diagnosis, paucibacillary tuberculosis, qPCR, saliva

## Abstract

**Background:**

Non-sputum-based tools are essential complements to the current tuberculosis diagnosis strategy. We aim to determine whether dual-gene qPCR melting curve detection of salivary exosomes *Mycobacterium tuberculosis* DNA can diagnose paucibacillary tuberculosis.

**Methods:**

We developed the exosomal nucleic acid (ExoNA)-based saliva tuberculosis diagnostic, a rapid, non-sputum diagnostic method for tuberculosis by integrating enrichment of salivary exosomes, nucleic acid extraction, and dual-target qPCR melting curve analysis. In a pilot clinical study, adults with suspected pulmonary tuberculosis were enrolled to evaluate diagnostic performance against multiple reference standards. Subgroup analysis was performed in patients with negative smear microscopy.

**Results:**

Salivary exosomal DNA was identified as the predominant form of detectable *Mycobacterium tuberculosis* DNA in saliva. ExoNA-based salivary tuberculosis diagnostic reduced the turnaround time from 16 h to 3h, and reached a limit of detection of 10 CFU/mL. In total, 130 patients with suspected pulmonary tuberculosis were included. Of them, 36·9% (48/130) were asymptomatic, and 80·8% (105/130) had negative smear microscopy results. The ExoNA-based Saliva TB assay had sensitivity of 81.2% (95% CI 53.7%-95.0%) and specificity of 82.8% (95% CI 63.5%-93.5%) against microbiological reference standards (n=45), while sensitivity of 68.4% (95% CI 54.6%-79.7%) and specificity of 82.9% (95% CI 67.4%-92.3%) under extended microbiological reference standards (n=98). In the subset of 105 patients with negative smear microscopy, the sensitivities remained within the range of 67.5% to 75.0% across different reference standards, and specificities ranged from 82.1% to 82.9%.

**Conclusion:**

ExoNA-based saliva tuberculosis assay demonstrates favorable diagnostic performance in patients with suspected pulmonary tuberculosis, with the advantages of non-invasive nature, easy sampling, and rapid detection. Multicenter validation in more heterogeneous populations is necessary prior to clinical use.

## 1 Introduction

It is estimated that 10·7 million people worldwide suffered from tuberculosis (TB) in 2024, but only 8·3 million were notified, with a substantial case detection gap (World Health Organization, 2025). Inadequate TB diagnostic capacity would limit patient detection, resulting in interpersonal transmission of TB. The World Health Organization (WHO) recommended the use of rapid molecular tests, such as the GeneXpert MTB/RIF (Xpert) (World Health Organization, 2013), for the initial diagnosis of TB, enabling timely initiation of treatment to improve treatment and clinical outcomes. However, these assays still face the challenge of sample collection among individuals with paucibacillary TB. Paucibacillary TB was defined as pulmonary TB with negative AFB smear results from respiratory specimens (Park et al., 2019). Notably, this form of TB poses considerable diagnostic challenges; as a result, invasive methods are often required to collect valid respiratory specimens, which may compromise patient adherence (Mondoni et al., 2017). Therefore, as indicated by the WHO, an alternative strategy under consideration is the use of non-sputum-based samples for diagnostic purposes (World Health Organization, 2014; Kasule et al., 2024).

Exosomes are cell-derived, vesicle-like structures secreted into the extracellular matrix (Kang et al., 2021). They encapsulate and transport a variety of biological cargo, including proteins, lipids, and nucleic acids, and mediate intercellular communication, maintenance of cellular homeostasis, and modulation of immune responses (Gurunathan et al., 2021). Given their abundance in body fluids, intrinsic stability, and enrichment in parental cell information, exosomes are recognized as a promising diagnostic marker (Zhou et al., 2020). A previous study detected virulence factors on blood-derived extracellular vesicles for sensitive diagnosis of pediatric TB, highlighting the potential of exosomes as diagnostic biomarkers for TB (Zheng et al., 2022). However, the nanoscale size of exosomes, along with contaminations from soluble proteins and lipoproteins, poses significant challenges for exosome isolation and purification, affecting their diagnostic application (Benayas et al., 2023). Extracting DNA from exosomes may bypass these challenges and facilitate its translation into clinical practice (Cho et al., 2020).

Saliva is easy to collect, exhibits good homogeneity, and is rich in exosomes (Wu et al., 2021). Salivary exosomes have been shown to share molecular features with those derived from plasma (Schmunk et al., 2025), which raises the hypothesis that TB-associated exosomes may also be present in saliva (Ayalew et al., 2024). Moreover, the lower biological complexity of the salivary matrix may facilitate the development of simplified exosome-based assays for TB (Katsani and Sakellari, 2019). This study aims to develop and validate a streamlined approach that integrates the efficient exosomal nucleic acid (ExoNA) enrichment with a high-sensitivity qPCR assay to detect *Mtb*-DNA from saliva.

## 2 Methods

### 2.1 Study design and participants

A diagnostic accuracy study was conducted to evaluate the clinical performance of the ExoNA-based saliva TB diagnostic. We prospectively enrolled patients with radiological findings suggestive of clinically suspected pulmonary tuberculosis (PTB) from April 1st, 2023, to September 30th, 2023. Eligible participants were > 15 years; negative for HIV infection; and had negative sputum smear results or were asymptomatic before hospital admission. Individuals with a prior history of TB or intolerance to bronchoscopy were excluded at enrollment. The study protocol was approved by the Ethical Review Committee of Shanghai Pulmonary Hospital (No. K21-257). Written informed consent was obtained from all participants. Notably, preprint data (Sha et al) covered the period before April 2023, while this study focuses solely on post-April samples, as we optimized the qPCR reaction system in April 2023, significantly boosting detection sensitivity (**Supplementary Information Table 1**). After deliberate consideration, we decided to only present the data after these key methodology improvements.

### 2.2 Study procedures

At baseline, all participants underwent a series of examinations, including clinical symptoms, QuantiFERON - TB (QFT) blood test, high-resolution chest CT scanning, mycobacterial smear and culture, as well as bronchoscopy. According to the X-ray localizer in chest CT, each lung was divided into three fields (upper, middle, and lower), resulting in a total of six regions (Petite Felipe et al., 2021). The number of infected lung fields was then evaluated. Two saliva samples were collected from each patient one day before sputum and bronchoalveolar lavage fluid (BALF) sample collection. 2 mL of saliva, obtained from sublingual or buccal secretions, were collected for the following ExoNA-based saliva TB testing. Sputum and BALF samples were tested for smear microscopy, BACTEC 960 culture, and Xpert assay. When sputum was unavailable, BALF would be submitted for the aforementioned tests alone. Positive cultures were then sent for species identification using the Bioline TB Ag MPT64 test (Abbott) to rule out the possibility of nontuberculous mycobacteria. All the tests were performed at the Shanghai Pulmonary Hospital. To avoid potential observer bias, the results from the ExoNA, Xpert, QFT, smears, and bacterial cultures were blinded until data analysis.

### 2.3 Collection and storage of saliva samples

Study participants were instructed to fast for 2 hours before saliva collection. Any activities that stimulate salivary gland secretion were prohibited. When sample collection, participants rinsed their mouths with water and rested for 2 minutes. Then, 2 mL of saliva was collected into a sterile disposable tube. Saliva samples could be stored at 20-25 °C for 2 hours, at 0-4 °C for up to 24 hours, and below -20 °C for 4 weeks to prevent DNA degradation. Samples that contain sputum or blood were considered unacceptable.

### 2.4 ExoNA-based Saliva TB diagnostic

**Figure 1:**
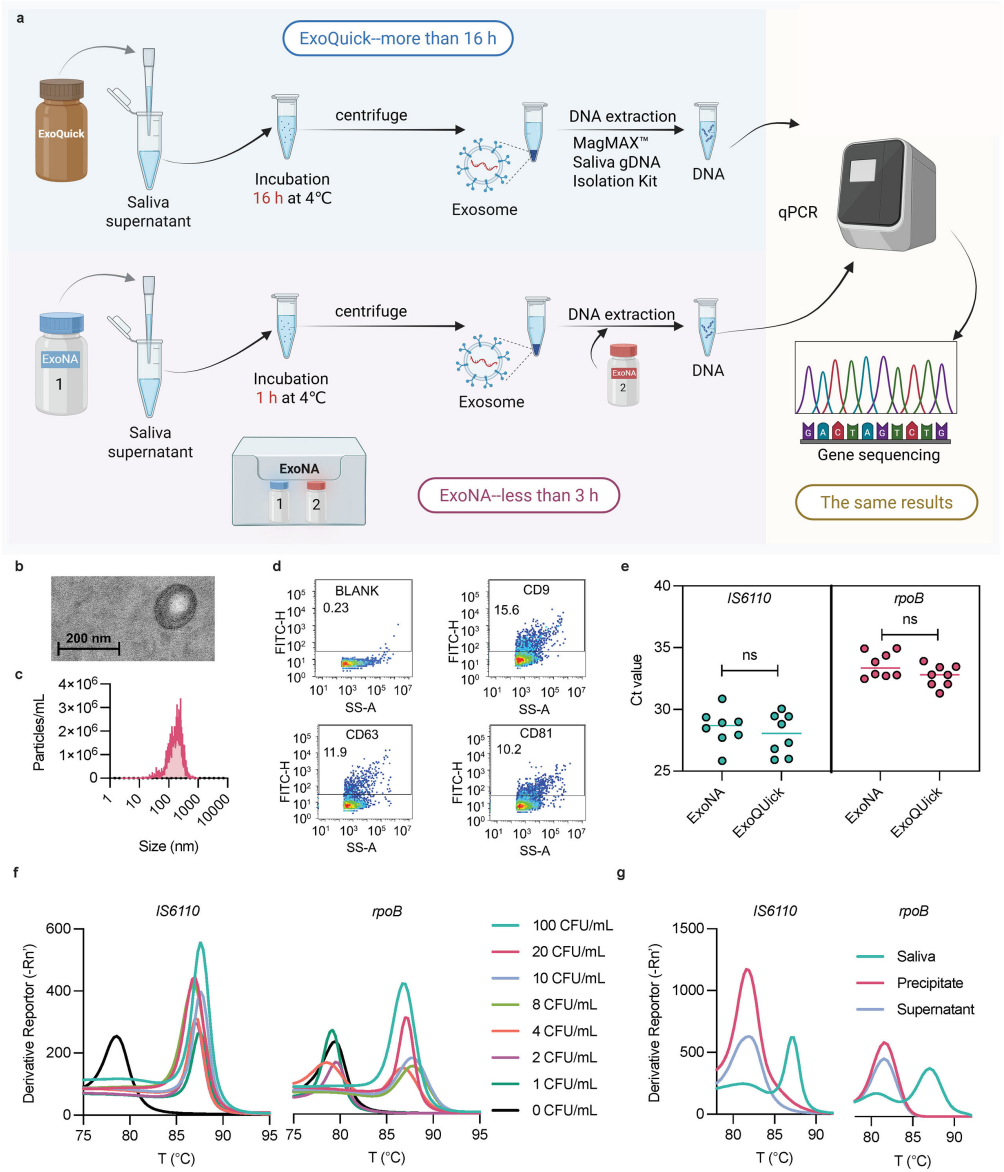
ExoNA-based Saliva TB diagnostic. Workflow for exosome isolation from saliva supernatant and subsequent DNA analysis using ExoQuick and ExoNA **(a)**. Transmission electron microscopy (TEM) images captured intact exosomal membranes with a clear bilayer structure **(b)**. Nanoparticle Tracking Analysis (NTA) of saliva - enriched exosomes showed particles sized between 50 nm and 150 nm, with a median diameter of ~100 nm and a concentration of approximately 4×10⁶ particles/mL. **(c)** Nanoparticle flow cytometry verified the exosomal proteins CD9, CD63, and CD81, confirming the isolation of exosomesb **(d)**. Dual-gene qPCR melting curve assay for exosomal DNA extracted via the ExoNA and MagMAX™ Saliva gDNA Isolation Kit methods. The Ct values of the two extraction methods ranged from 31 to 35, with no statistically significant difference observed between them (Paired t test, *P* > 0.05) **(e)**. Using a dual-gene qPCR melting curve assay, *IS6110* and *rpoB* were successfully detected, with minimum detectable concentrations of 10 CFU/mL and 40 CFU/mL, respectively, from the *MTB* H37Rv strain **(f)**. To validate the origin of *MTB* genes in saliva, DNA was extracted from salivary supernatant, whole saliva, and precipitate. Only the supernatant tested positive, while whole saliva and precipitate yielded negative results in qPCR analysis **(g)**.

The ExoNA-based saliva TB diagnostic employs a three-step approach: exosome isolation and nucleic acid extraction using the Mediated Salivary Exosome Enrichment Kit (Shanghai Liquidbio Bio-tech Co., Ltd.; currently in joint R&D phase with our team), followed by the detection of *Mtb*–specific genes (*IS6110* and *rpoB*) using a dual-gene qPCR melting curve assay (DGPMC) (**Figure 1a**). In brief, 280 μL of saliva samples were initially centrifuged at 3000 g for 10 minutes. Then, the supernatant was mixed with 420 μL exosome precipitation solution (kit components 1) and incubated on ice or at 4 °C for 1 hour. The resulting mixture was subsequently centrifuged at 3000 g for 10 minutes to precipitate the exosomes. Then, 700 μL of lysis buffer and magnetic beads (kit components 2) were added to resuspend the sediment from the last step. After gently inverted and shaken for 15 minutes, the magnetic beads absorbed with exosomal DNA were separated and washed twice with 75% ethanol and eluted with 60 μL of TE buffer.

The eluted DNA was tested as previously reported (Ma et al., 2025): 22 μL of DNA was added to the 28 μL master mix (Buffer; dNTP/dUTP; DNA polymerase; SYBR Green; Primers), followed by UNG treatment at 25 °C for 10 min to prevent contamination, Taq activation at 95 °C for 3 min, 40 cycles of 95 °C for 10 s and 58 °C for 40 s with fluorescence detection during extension, and a post-amplification melting curve analysis to confirm product specificity. A sample was considered positive if it met both of the following conditions: 1) Ct values for *IS6110* < 30 or *rpoB* < 35; 2) melting curve temperatures of *IS6110* and *rpoB* were between 86·0 °C and 88·2 °C (Ma et al., 2025). If one of the genes meets both of the above conditions, the result can be determined as positive.In the melting curve method, a single plate allows simultaneous detection of 93 reaction wells (corresponding to 46 clinical samples), 1 positive control, 1 negative control, and 1 internal standard control via a fluorescence quantitative PCR instrument.

To evaluate the efficiency of exosome and exosomal nucleic acid extraction, the commercial exosome enrichment kit (ExoQuick, System Biosciences) and the MagMAX™ Saliva gDNA Isolation Kit (Cat. No. A39060) were used as controls (**Figure 1a**).

### 2.5 Definitions for reference standards

We applied the definition for multiple reference standards according to the latest guidelines (Drain et al., 2019). For the microbiological reference standard (MRS), participants with at least one positive result in *Mtb* sputum culture or Xpert assay are classified as TB cases. For the extended microbiological reference standard (eMRS), confirmed PTB cases were defined as at least one positive result of Xpert or *Mtb* culture from sputum and BALF samples. The TB-negative cases were defined as none of the positive results were found on Xpert nor *Mtb* culture from sputum or BALF, and QFT results were negative.

Regarding clinical TB standards, those who did not have a positive culture or Xpert result, and the decision to treat for TB was made by the clinician, were defined as clinically diagnosed TB. Meanwhile, they should meet at least one of the following: positive smear from BALF, chest CT scan suggestive of PTB, or efficient response to antituberculosis treatment (World Health Organization, 2013). Patients with other pulmonary diseases were defined as those having definite pathological or bacteriological evidence of pulmonary diseases but without positive results from Xpert, mycobacterial culture, and QFT tests. As for excluding the clinical TB standard, clinically diagnosed TB patients are excluded from the analysis. Unclassifiable group consisted of those who were microbiologically and clinically negative of TB but having positive QFT result.

### 2.6 Statistical analysis

Continuous variables were summarized as median and interquartile ranges (IQR), and categorical variables as counts and percentages. The sensitivity and specificity of ExoNA-based saliva TB assay was evaluated against reference standards and expressed with 95% confidence intervals (CIs). All data analyses were performed with forestplot package (version 3·1·7) in R (version 4·3·2) and GraphPad Prism (version 8·0·2), and the flow chart was prepared using BioRender.

## 3 Results

### 3.1 ExoNA-based Saliva TB diagnostic

We identified saliva-derived exosomes using nanoparticle flow cytometry and transmission electron microscopy (**Supplementary Information S2**). As shown in **Figures 1b–d**, exosome-specific proteins CD9, CD63, and CD81 were detected, and the vesicles exhibited a distinct bilayer structure. The nanoparticle tracking analysis revealed the particle sizes ranging from 50 to 150 nm, with a median diameter of 100 nm and a particle concentration of 5·0 × 10^6^/mL. The exosomal DNA extracted by the ExoNA and MagMAX™ Saliva gDNA Isolation Kit both showed high purity with OD260/OD280 ratios above 1·8. Using a dual-gene qPCR melting curve assay, this detection method can successfully detect the exosomal DNA extracted with the ExoNA Kit and the MagMAX™ Saliva gDNA Isolation Kit. Moreover, the Ct values of these two extraction methods ranged from 31 to 35, with no statistically significant difference observed between them (Paired t test, *P* > 0.05) (**Figure 1e**). Additionally, this assay enabled the successful detection of IS6110 and rpoB from the *Mtb* H37Rv strain, with a minimum detectable concentration of 10 CFU/mL (**Supplementary Information S3**; **Figure 1f**). Overall, the performance of exosomal DNA extracted by ExoNA was comparable to that of the commercial ExoQuick approach, with a much shorter turnaround time (<1 hour versus >16 hours).The full workflow, including exosome purification, DNA purification and qPCR analysis, was around 3 hours. (**Figure 1a**).

To evaluate the capacity of the ExoNA-based Saliva TB diagnostic in detecting *Mtb* from clinical samples, four saliva samples collected from patients with sputum culture-confirmed TB were tested. The ExoNA assay yielded complete positive results, whereas the smear, culture, and Xpert assay all returned negative results, highlighting the high sensitivity of ExoNA assay, which may be contributed the exosome-mediated target concentration effect.

To validate this hypothesis, 280 μL samples of whole saliva (without prior supernatant separation) and supernatant and sediment were analyzed using the ExoNA in 10 patients with microbiologically confirmed PTB. The results showed that 80% (8/10) of the supernatant samples were positive while only 10% (1/10) of the whole saliva and sediment samples were positive. (**Figure 1g**; **Table 1**). These findings supported our hypothesis that the detectable fraction of *Mtb* DNA in saliva primarily from exosomes in the supernatant, and that exosome-derived DNA substantially reduces interference from endogenous inhibitors and host-derived nucleic acids, thereby improving diagnosis performance.

**Table 1:** Analysis of DNA signal origin across salivary components by targeting the *rpoB* and *IS6110* gene.

Sample No·	Saliva supernatant					Saliva precipitate					Whole saliva				
	*IS6110*		*rpoB*		qPCR result	*IS6110*		*rpoB*		qPCR result	*IS6110*		*rpoB*		qPCR result
	Ct	Tm	Ct	Tm		Ct	Tm	Ct	Tm		Ct	Tm	Ct	Tm	
**1**	29·30	86·86	32·15	/	+	28·94	86·86	35·93	/	+	29·25	86.67	30·60	87·60	+
**2**	27·73	87·05	31·16	88·27	+	27·.52	/	32·18	/	–	27·85	/	33·71	/	–
**3**	28·89	/	33·95	/	–	29·02	/	35·30	/	–	27·36	/	31·43	/	–
**4**	30·14	87·24	31·06	87·76	+	27·62	/	30·60	/	–	27·48	/	32·94	/	–
**5**	29·37	86·93	33·22	/	+	34·19	/	36·56	/	–	33·71	/	30·69	/	–
**6**	33·60	/	30·38	87·76	+	28·66	/	38·43	/	–	28·27	/	33·02	/	–
**7**	36·25	/	33·22	/	–	31·20	/	33·79	/	–	30·60	/	32·50	/	–
**8**	28·63	87·24	32·85	/	+	30·81	/	27·47	/	–	31·02	/	32·79	/	–
**9**	27·49	87·61	30·60	87·60	+	30·94	/	30·97	/	–	30·73	/	28·63	/	–
**10**	29·42	87·61	30·19	87·76	+	27·35	/	35·85	/	–	26·51	/	34·95	/	–
**Rate**	**80·0%**					**10·0%**					**10·0%**				

### 3.2 Pilot diagnostic accuracy trial

**Figure 2:**
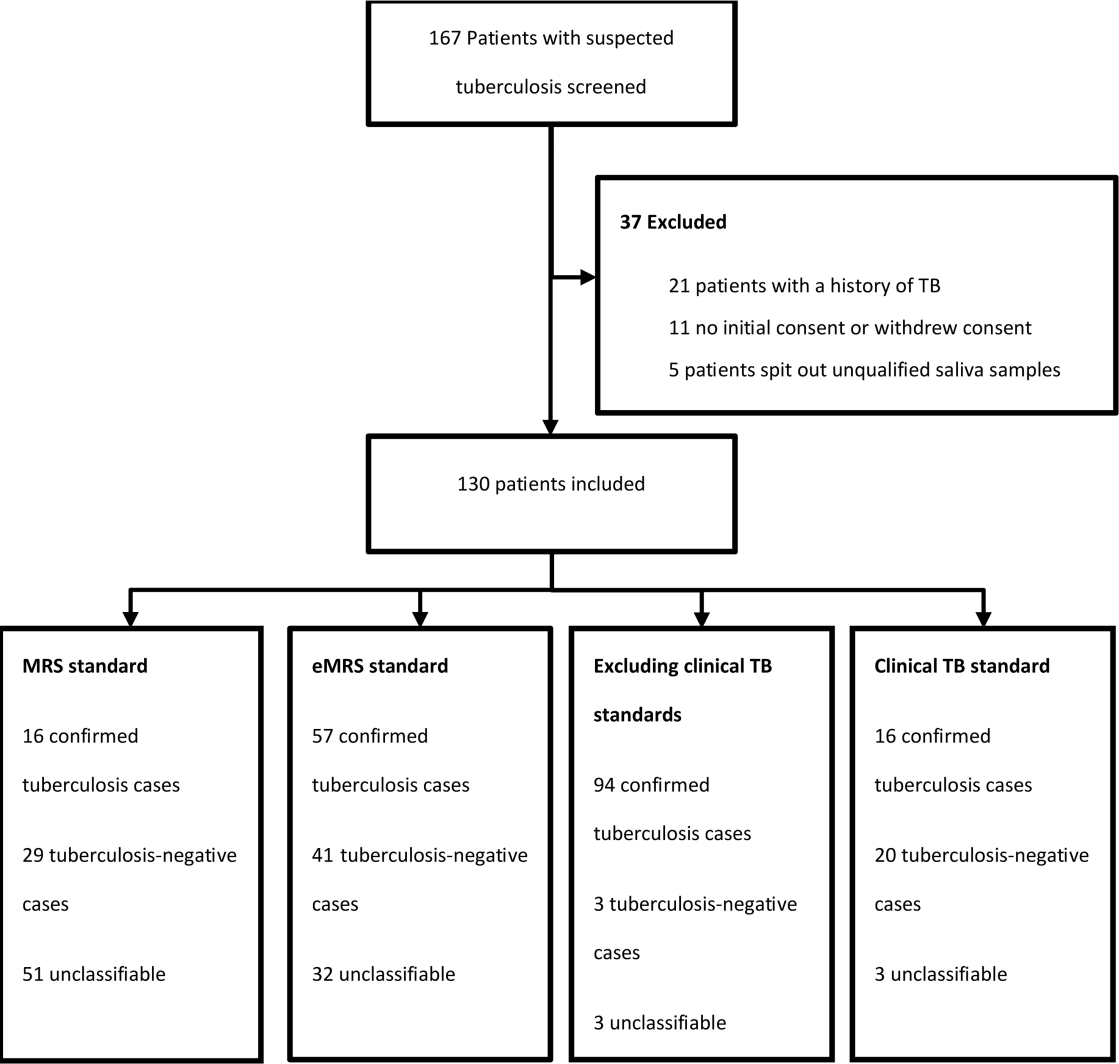
Participant flow, exclusions from analytical eligibility, and TB-status populations stratified by reference standards. Four reference standards are defined as follows: ① Microbiological Reference Standard (MRS): PTB cases with at least one positive result in Mtb sputum culture or Xpert assay. ② Extended Microbiological Reference Standard (eMRS): Confirmed PTB with at least one positive result in Xpert or Mtb culture of sputum/BALF. ③ Excluding Clinical TB Standard: Analyses excluding clinically diagnosed PTB cases. ④ Clinical TB standards: Clinically diagnosed PTB is defined as no positive culture or Xpert result, plus at least one of the following: positive BALF smear, chest CT suggestive of PTB, or effective anti-TB treatment response.

A total of 167 patients were screened during the study period, 37 patients were excluded due to the infection history of TB and other reasons. Finally, 130 patients were included for analysis with a median age of 49·5 (IQR: 32·0-59·0) years and 60% being male. (**Figure 2**) The number of patients being asymptomatic or having comorbidities were 48 (36·9%) and 15 (11·5%) respectively. The median lung infected areas were 1 (IQR: 1-2). Negative sputum acid-fast staining results were reported for 105 patients (80·8%). The rates of positive culture for sputum and BALF samples were 12·3% and 28·5%, respectively (**Table 2**). Under the four distinct diagnostic criteria, the number of cases in the unclassifiable group was 51, 32, 3, and 3, respectively.

**Table 2:** Patient characteristics and laboratory results.

	Patients suggestive of tuberculosis (n=130)
Age, years	49·5 (32·0, 59·0)
Sex	
Men	78 (60·0)
Women	52 (40·0)
Tuberculosis suggestive symptoms	
Cough	62 (47·7)
Expectoration	52 (40·0)
Fever	21 (16·2)
Haemoptysis	13 (10·0)
Chest pain	15 (11·5)
No symptoms	48 (36·9)
Comorbidity	
COPD	5 (3·8)
Diabetes mellitus type 2	10 (7·7)
Chest CT scan	
No· of infected pulmonary zones	1 (1, 2)
Pulmonary cavity	20 (15·4)
QuantiFERON-TB	
Positive	82 (63·1)
Negative	47 (36·2)
Not done	1 (0·8)
Smear microscopy (Sputum)	
4+	0 (0)
3+	2 (1.5)
2+	3 (2·3)
1+	4 (3·1)
Negative	105 (80·8)
Not done	15 (11·5)
GeneXpert (Sputum)	
High	0 (0)
Medium	3 (2·3)
low	0 (0)
Very low	0 (0)
Negative	25 (19·2)
Not done	102 (78·5)
MGIT culture (Sputum)	
Positive	16 (12·3)
Negative	74 (56·9)
NTM	5 (3·8)
Not done	35 (26·9)
Smear microscopy (BALF)	
4+	1 (0·8)
3+	1 (0·8)
2+	4 (3·1)
1+	13 (13·0)
Negative	110 (84·6)
GeneXpert (BALF)	
High	3 (2·3)
Medium	12 (9·2)
low	10 (7·7)
Very low	24 (18·5)
Negative	81 (62·3)
MGIT culture (BALF)	
Positive	37 (28·5)
Negative	89 (68·5)
NTM	4 (3·1)
ExoNA (saliva)	
Positive	58 (44·6)
Negative	72 (55·4)

Data are presented as median (IQR) or n (%). Each lung was divided into upper, middle, and lower zones based on chest CT localization, yielding a total of six pulmonary regions. Infected zones were defined as lung parenchymal areas with typical tuberculous CT findings (e.g., consolidation, ground-glass opacity, cavitation, or nodules), while uninfected zones showed no such imaging abnormalities.

**Figure 3:**
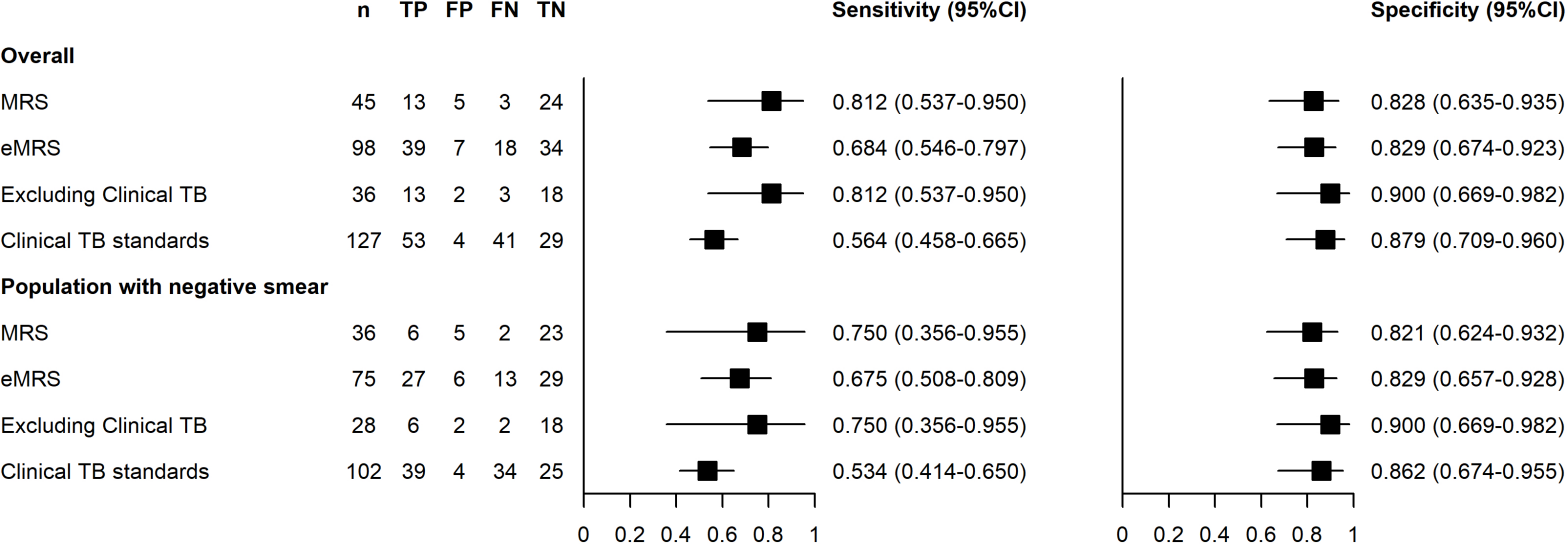
Assessment of ExoNA-based saliva TB Method's Sensitivity and Specificity with Multiple Reference Standards.

Under excluding clinical TB standard, the sensitivity of the ExoNA-based Saliva TB assay was 81·2% (13/16, 95% CI 53·7%-95·0%) and the specificity was 90·0% (18/20, 95% CI 66·9%-98·2%). When against the MRS, the sensitivity was 81·2% (13/16, 95% CI 53·7% - 95·0%), and the specificity was 82·8% (24/29, 95% CI 63·5%-93·5%). The sensitivity decreased to 68·4% (39/57, 95% CI 54·6%-79·7%) under eMRS, while the specificity remained to be 82·9% (34/41, 95% CI 67·4%-92·3%). Regarding clinical TB standard, the sensitivity further reduced to 56·4% (53/94, 95% CI 45·8%-66·5%) and the specificity slightly increased to 87·9% (29/33, 95% CI 67·4%-96·0%). (**Figure 3**).

In the study cohort of negative smear acid-fast staining (total 105 patients), compared to Excluding Clinical TB standard, the sensitivity of the ExoNA-based Saliva TB assay was 75·0% (6/8, 95% CI 35·6%-95·0%) and the specificity was 90·0% (18/20, 95% CI 66·9%-98·2%). Compared to MRS standard, the sensitivity was75·0% (6/8, 95% CI 35·6%-95·0%), and the specificity was 82·1% (23/28, 95% CI 62·3% - 93·2%). Compared to eMRS standard, the sensitivity was 67·5% (27/40, 95% CI 50·8%-80·9%) and the specificity was 82·9% (29/35, 95% CI 65·7%-92·8%). Compared to clinical TB standard, the sensitivity was 53·4% (39/73, 95% CI 41·4%-65·0%) and the specificity was 86·2% (25/29, 95% CI 67·4%-95·5%) (**Figure 3**). The number of cases with positive ExoNA-based Saliva TB assay results in the four unclassifiable subgroups was 27, 13, 1, and 1, respectively.

## 4 Discussion

In this study, we developed a novel assay for TB detection, which was based on the dual-gene qPCR technique and targeted the *Mtb*-specific genes from salivary exosome. The exosome was effectively enriched and isolated within three hours, and the characteristics and origin were verified. In pilot diagnostic accuracy trial, the performance of ExoNA-based Saliva TB assay was assessed to be promising with sensitivities ranging from 68·4% to 81·2% and specificities ranging from 82·8% to 90·0% across different microbiological reference standards, and robust in the population with negative sputum smear. Our findings indicate that this novel assay has the potential for clinical translation, especially in those with paucibacillary TB or being asymptomatic.

The performance of ExoNA in exosome enrichment and isolation was comparable to the commercial ExoQuick method. As previous study showed, the ExoQuick exosome extraction protocol is distinguished by its operational simplicity and efficiency, obviating the need for ultracentrifugation while demonstrating broad applicability to diverse biological fluids (Veerman et al., 2021). It has been used as the optimal method for exosome isolation in the diagnosis of Parkinson’s disease (Nila et al., 2022). In our study, we made full advantage of prior developed dual-gene qPCR melting curve method with a detection limit of 10 CFU/m L, which is specifically optimized for patients with paucibacillary TB (Ma et al., 2025). With this method, the ExoNA could achieve high DNA purity (OD260/OD280 > 1·8). In addition to comparable performance with the ExoQuick, the ExoNA method has the advantage of much shorter turnaround time. ExoNA’s optimized 3-hour TB diagnostic process features non-invasiveness, convenience, and accuracy in low bacterial load populations, and is configured for high-volume settings with no manual supervision post-reagent addition. Saliva’s lower complexity simplifies exosome assays to boost scalability, while integrated purification eliminates protein/host DNA interference, ensuring robust sensitivity and specificity for hard-to-detect paucibacillary TB.

The sensitivity and specificity of ExoNA were shown to be decent in *Mtb* detection. In 2015, WHO reviewed and recommended the first non-sputum biomarker test for TB, the urinary lipoarabinomannan (FujiLAM) assay, which showed a sensitivity of 42% and a specificity of 91% in a meta-analysis (Bjerrum et al., 2019). Another study used FujiLAM and AlereLAM in the urine of HIV-positive patients with symptoms of TB and proved the higher sensitivity of FujiLAM (60% vs. 40%) (Huerga et al., 2023). A diagnostic accuracy study using triple-gene host-response reagents for childhood TB showed a sensitivity of 59·8% against microbiological criteria and 29·6% against clinically compliant reference criteria, with a specificity of 90·3% against both criteria (Olbrich et al., 2024). Compared with the aforementioned well-validated non-sputum diagnostic methods, the performance of ExoNA is comparable to the latter, even though their detection principles and target populations are distinct. Under microbiological reference standards, it shows even better sensitivity. The specificity of ExoNA still does not meet the benchmark advocated by the WHO for TB diagnostic assays (Drain et al., 2019). Cases missed by traditional assays were flagged positive by ExoNA, some of which may be classified as false positives under non-clinical TB standards. Specifically, ExoNA exhibited approximately 82% specificity against MRS/eMRS standards, a figure that rose to >88% when clinical TB standards were applied. This shift supports that the observed specificity range may not be an assay limitation, but rather its capacity to detect cases overlooked by conventional approaches.

Robust performance of ExoNA in those with paucibacillary TB was observed in subgroup analysis. Given that incipient TB is not detectable on the basis of symptoms, microbiological testing (Drain et al., 2018), the sensitivity of traditional methods were usually not high, especially in patients with low bacterial load. As novel TB screening and diagnostic tools, tongue swab sampling combined with qPCR for the detection of *MTB* DNA emerges as a promising alternative to conventional sputum-based testing for TB diagnosis (Olson et al., 2024). A recent report by Andama has also found that tongue swab tests remained negative for samples with marginal (“trace”) Ultra positivity in sputum analysis (Andama et al., 2022). Although ExoNA exhibited a mild decline in diagnostic efficacy among patients with smear-negative results in our cohort, this reduction does not undermine its potential as an adjunctive tool for paucibacillary TB diagnosis. Notably, its robust performance under low bacterial load conditions stems from the optimized exosome enrichment-purification-lysis protocol—a procedure that specifically enriches salivary exosomes, yet does not target *MTB* nucleic acids that merely settle in the oropharynx via respiratory shedding.

We observed an interesting finding: among the uncategorized patients defined by clinical TB diagnostic standards, those who tested positive for QFT but were excluded from active tuberculosis were considered to have latent tuberculosis infection (LTBI). Some of them showed positive ExoNA results, likely due to the method’s high sensitivity. Previous reports have demonstrated that DNA fragments of *Mtb* can be detected in the body fluids of patients with LTBI (Mayito et al., 2020). For this reason, we excluded these individuals in this study, which may limit the generalizability of our findings and restrict the applicability to broader populations. Nevertheless, the promising results of this pilot study support continued efforts to refine the method and enhance its ability to distinguish between LTBI and active TB disease.

This study has some limitations. Firstly, it was carried out at a single center, which potentially limits the generalization of study findings. Secondly, the diagnostic specificity of ExoNA was slightly lower than the requirements for non-sputum tests released by the WHO (Drain et al., 2019). However, it should be noted that the majority of patients included in this study were carriers with low bacterial loads and presented with mild symptoms. The low bacteriological level in the samples may lead to false-negative culture outcomes, which in turn causes the “false positivity” for ExoNA. Accordingly, future multicenter clinical studies will incorporate a more diverse range of cases and further explore changes in the new method’s diagnostic sensitivity across the spectrum of Xpert Ct values in patients with different bacterial loads.

The active identification of PTB patients represents a principal measure in the establishment of a TB-free community (Xu and Zhao, 2025) and a core component of global efforts to achieve the WHO’s End TB Strategy goals. ExoNA assay, leveraging its merits of non-invasive saliva sampling and rapid on-site detection, is primarily intended for patients suspected of tuberculosis, close contacts of TB patients, and even the general healthy population, as it advances screening thresholds to facilitate prompt detection of incipient TB and early risk stratification of suspected populations in both clinical and community settings. In the future, we aim to conduct comprehensive multi-center validation studies across diverse populations, further optimize the assay’s performance, and utilize this test for the accurate diagnosis of extrapulmonary tuberculosis as well as the precise identification and differentiation of LTBI from active TB infection.

In conclusion, ExoNA detection shows good sensitivity and specificity for TB detection in the patients with suspected PTB. Considering its advantages of being easy to operate, non-invasive, enabling repeatable sample collection, having satisfactory sensitivity and specificity, as well as a short detection time, ExoNA may provide a good supplement to traditional TB detection methods. This is especially the case when it comes to detecting TB infection in suspected TB patients who have a low bacterial load and exhibit no or mild clinical symptoms. To validate its robustness, a multicenter study in more heterogeneous populations is required prior to clinical application.

## Data Availability

The original contributions presented in the study are included in the article/**Supplementary Material**, further inquiries can be directed to the corresponding author/s.
