# Correction: Salivary exosomal *Mycobacterium tuberculosis* DNA enables sensitive detection of paucibacillary tuberculosis: a molecular diagnosis study

**DOI:** 10.3389/fcimb.2026.1930665

**Published:** 2026-08-03

**Authors:** Jun Ma, Xubin Zheng, Yifan He, Xuewen Tang, Zhen Huang, Li Wang, Xing Tang, Yuanyuan Huang, Zhizhi Lei, Min Zhu, Wei Sha

**Affiliations:** 1 Department of Tuberculosis, Shanghai Pulmonary Hospital, School of Medicine, Tongji University, Shanghai, China; 2 Clinic and Research Center of Tuberculosis, Shanghai Pulmonary Hospital, School of Medicine, Tongji University, Shanghai, China; 3 Division of Infectious Diseases, Department of Medicine, Karolinska Institute, Stockholm, Sweden; 4 Department of Infectious Diseases, Karolinska University Hospital, Stockholm, Sweden; 5 Shanghai Liquidbio Bio-tech Co., Ltd., Shanghai, China; 6 Center for Cellular and Molecular Diagnostics, Tulane University School of Medicine, New Orleans, LA, United States; 7 Department of Medicine, Tulane University of Medicine, New Orleans, LA, United States; 8 Shanghai Key Laboratory of Tuberculosis, Shanghai Pulmonary Hospital, School of Medicine, Tongji University, Shanghai, China

**Keywords:** exosome, molecular diagnosis, paucibacillary tuberculosis, qPCR, saliva

Author “Xubin Zheng” was erroneously omitted as equal contributing author. The original version of this article has been updated.

