# Supplementary material for: Salivary exosomal Mycobacterium tuberculosis DNA enables sensitive detection of paucibacillary tuberculosis: a molecular diagnosis study
Source: Front Cell Infect Microbiol. 2026 Jun 1;16:1811140. doi: 10.3389/fcimb.2026.1811140 (PMC13265331; doi:10.3389/fcimb.2026.1811140)
Supplement: Supplementary file 1 [file Table1.docx]

**Contributors**

Conception and design of the study: WS and JM. Acquisition of data: YH, LW, XT and JM. Analysis and interpretation of data: LW, JM, XT, ZL, MZ, HY and XZ. Drafting the article: YH, JM and XZ. Revising it critically for important intellectual content: YH, JM, ZH, XZ, and WS. Final approval of the version to be submitted: all authors.

Contributions made by the industry for each author.

**Data sharing**

De-identified participant data will be made available upon requests directed to the chief investigator. Proposals will be reviewed by the sponsor, study team, chief investigator, and collaborators on the basis of scientific merit and a response can be expected within 14 days. Requests should be made to the corresponding author (shfksw@tongji.edu.cn). After approval of a proposal, data can be shared through a secure online platform after signing a data access agreement.

**Declaration of interests**

All authors declare no competing interests.

**Acknowledgments**

We acknowledge the support of Shanghai Liquidbio Biotechnology Co., Ltd. for providing support in experimental technologies for this study.

**Funding sources**

This work was supported by the National Key R&D Program of China (2023YFC2307300, 2023YFC2307305); National Science and Technology Major Project on Prevention & Control of Emerging, Re-emerging & Major Infectious Diseases (2025ZD01907101); Risk Prediction of Onset and Precision Intervention Strategies for Populations Infected with Major Chronic Infectious Diseases (25Y32800100); National Natural and Science Foundation of China (82302614); Shanghai three-year (2023-2025) action plan to strengthen the public health system (GWVI-11.2-YQ06). The funders played no role in the study design, data collection, analysis, interpretation, or manuscript writing.

**Supplementary appendix**

This appendix formed part of the original submission and has been peer reviewed.

We post it as supplied by the authors.

Supplement to: Salivary exosomal *Mycobacterium tuberculosis* DNA enables sensitive detection of paucibacillary tuberculosis: a molecular diagnosis study

**Contents**

Supplementary S1 Key reagents 3

Supplementary S2 Verification of Salivary Nucleic Acids Derived from Exosomes 4

Supplementary S3 Supplementary Experiment on the Limit of Detection of Dual-Gene qPCR Melting Curve Assay (DGPMC) 4

[Supplementary table 1 5](#_Toc197613008)

[Supplementary table 2 6](#_Toc197613008)

[Supplementary Figure 1 7](#_Toc197613008)

[Supplementary Figure 2 7](#_Toc197613008)

[Supplementary Figure 3 8](#_Toc197613008)

# Key reagents

DNA polymerase (Cat. No. 13689ES76) and qPCR SYBR Green Master Mix (Cat. No. 11202ES08) were purchased from YEASEN Biotechnology Co., Ltd. (Shanghai, China). TE buffer (Cat. No. B5410190100) and nuclease-free water (Cat. No. A5001970500) were obtained from Sangon Biotech Co., Ltd. (Shanghai, China). ExoNA Exosome Concentration Solution (ECS) and the DNA Extraction Kit (Cat. No. LS2101; Supplementary Table 2) were obtained from Shanghai Liquid-Bio Biotechnology Co., Ltd. The *Mycobacterium tuberculosis* H37Rv strain was obtained from the NIH. ExoQuick (Cat. No. EXOQ20a-1) was purchased from System Biosciences, LLC.

# Verification of Salivary Nucleic Acids Derived from Exosomes

In brief, 280 μL of saliva samples were initially centrifuged at 3000 g for 10 minutes. Then, the supernatant was mixed with 420 μL exosome precipitation solution (kit components 1) and incubated on ice or at 4°C for 1 hour. After incubation, the sample is centrifuged at 3000rpm for 10 minutes. A white precipitate appears in the tube, which contains exosomes (Supplementary Figure 1). Dissolve the precipitate in 50 μL of 0.1 M phosphate-buffered saline (PBS), and transport it on ice for exosome nano-flow cytometry and nanoparticle tracking analysis (NTA). The white precipitate was fixed in 2% glutaraldehyde for 12–48 hours, followed by post-fixation in 1% osmium tetroxide (OsO₄) for 2 hours. Subsequently, gradient dehydration was performed using 30%–100% ethanol, and the sample was embedded in epoxy resin. Following resin polymerization, the exosome-embedded epoxy resin blocks were sectioned into 50–70 nm-thick ultrathin sections using an ultramicrotome, which were then observed under a transmission electron microscope (TEM).

qPCR is used to detect the housekeeping gene *GAPDH* in both the polymer precipitate and residual fluid to verify that salivary DNA is derived from exosomes.

Using DNA extracted from the polymer precipitate by Component 2 of the ExoNA as a template, qPCR analysis of *GAPDH* yielded a mean Ct value of approximately 30·7. Using DNA extracted from the residual fluid after removing the Component 2 of The ExoNA as a template, qPCR detection of *GAPDH* showed no CT value. This experiment was repeated three times, with consistent results each time (Supplementary Figures 2).

This experiment indicates that after saliva sample processing with The ExoNA, exosomal nucleic acids are mainly in the precipitate, and the residual fluid after precipitation removal contains little or no exosomal nucleic acids.

This experiment indicates that after saliva sample processing with The ExoNA, exosomal nucleic acids are mainly in the precipitate, and the residual fluid after precipitation removal contains little or no exosomal nucleic acids.

# Supplementary Experiment on the Limit of Detection of Dual-Gene qPCR Melting Curve Assay (DGPMC)

For the establishment of the detection limit, it is necessary to conduct at least 20 repeated tests at the detection limit concentration level, and the positive detection rate should reach 95% to be more scientific. Therefore, the experimental data are supplemented.

The minimum detection limit concentration of *IS6110* gene was 10 CFU/mL, and the positive detection rate was 100% after 20 repeated experiments. Similarly, the minimum detection limit concentration of *rpoB* was verified 20 times at 40 CFU/mL, and the positive detection rate was 100%. (Supplementary Figures 3)

**Supplementary tables 1 Around April 2023: Optimization and Adjustment of the qPCR**

| The optimized reaction system after April 2023 | | | The reaction system before April 2023 | | | |
| --- | --- | --- | --- | --- | --- | --- |
| Reagent | Volume（μL） | Final conc. | Reagent | Volume（μL） | Final conc. |  |
| ArtiCanATM SYBR qPCR Mix-UDG (Low ROX Premixed) | 25 | 1ⅹ | Hieff® qPCR SYBR Green Master Mix (Low Rox Plus) | 15 | 1ⅹ |  |
| *IS6110/rpoB* Forward Primer(10μM) | 1 | 0.1 μM | Uracil DNA Glycosylase(UDG),heatlabile (1 U/μL) | 0.6 | 0.02 U/μL |  |
| *IS6110/rpoB* Reverse Primer (10μM) | 1 | 0.1 μM | *IS6110/rpoB* Forward Primer(10μM) | 0.6 | 0.2 μM |  |
| *GAPDH* Forward Primer (10μM) | 0.4 | 0.2 μM | *IS6110/rpoB* Reverse Primer (10μM) | 0.6 | 0.2 μM |  |
| *GAPDH* Reverse Primer (10μM) | 0.4 | 0.2 μM |  |  |  |  |
| Sample | ≤22 | / | Sample | ≤13 | / |  |
| Add ddH_2_O to total volume | 50 | / | Add ddH_2_O to total volume | 30 |  |  |

**Note:** After optimization of the PCR system, the detection sensitivity was improved. The minimum detection limit (MDL) of the *rpoB* gene was reduced from approximately 320 CFU/mL to 40 CFU/mL, and that of the *IS6110* gene was reduced from approximately 40 CFU/mL to 10 CFU/mL.

# Supplementary tables 2 The ExoNA kit formulation.

| Component 1 | Proportion (%) | Function |
| --- | --- | --- |
| Exosome Precipitation Solution | 5 | Selectively precipitate exosomes by spatial exclusion and dehydration effects. |
| Component 2  Combination of lysis buffer and magnetic beads | Proportion (%) |  |
| LiCl | 0.5 | nucleases inhibition helper |
| Proteinase K | 1 | Degrade proteins and eliminate nuclease activity |
| Guanidine thiocyanate | 2.5 | Strong denaturants, destruction biofilms and nucleases;separate nucleic acid from protein |
| Trisodium citrate | 3 | Inhibition of metal ion-dependent nucleases; PH buffer |
| Sodium N-lauroylsarcosinate | 2 | Destruction biofilms and nucleases |
| 2-mercaptoethanol | 1 | Inhibit RNase activity; prevent nucleic acid oxidative damage |
| Isopropanol | 1 | Reduce the solubility of nucleic acids and precipitation helper |
| EDTA | 0.5 | Chelator, inhibition of metal ion-dependent nucleases |
| nuclease- free water | 90 | Provide a nuclease-free environment |

# Supplementary Figure


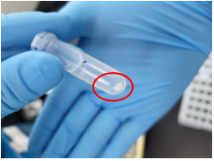


**Supplementary Figure 1**

The white precipitate contains exosomes required for detection.


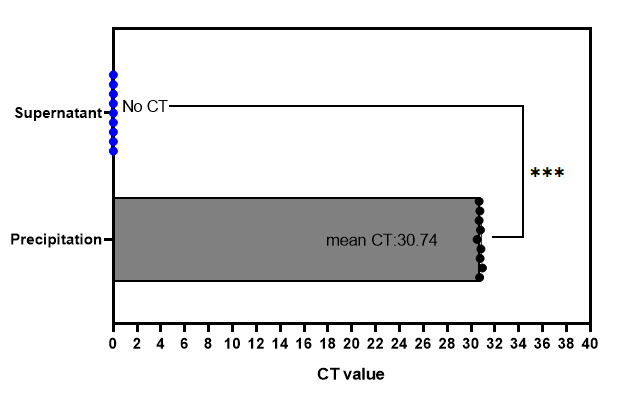


**Supplementary** **Figure 2**

qPCR Ct values of *GAPDH* in the residual fluid (Supernatant) and polymer precipitate (extracted via ExoNA Component 2). The polymer precipitate showed a mean *GAPDH* Ct value of 30.74, while no Ct value was detected in the residual fluid. Results were consistent across 3 experimental replicates (*** indicates significant difference).

**Supplementary Figure 3**

The minimum detection limit concentration of *Mycobacterium tuberculosis* *IS6110* gene was 10 CFU/mL, and the positive detection rate was 100% after 20 repeated experiments, as shown in Fig. 3a. Similarly, the minimum detection limit concentration of *Mycobacterium tuberculosis* *rpoB* was verified 20 times at 40 CFU/mL, and the positive detection rate was 100%, as shown in Fig. 3b.
