# Supplementary material for: Salivary exosomal Mycobacterium tuberculosis DNA enables sensitive detection of paucibacillary tuberculosis: a molecular diagnosis study
Source: Front Cell Infect Microbiol. 2026 Jun 1;16:1811140. doi: 10.3389/fcimb.2026.1811140 (PMC13265331; doi:10.3389/fcimb.2026.1811140)
Supplement: Supplementary file 2 [file Table2.docx]

**Supplementary appendix**

This appendix formed part of the original submission and has been peer reviewed.

We post it as supplied by the authors.

Supplement to: Salivary exosomal *Mycobacterium tuberculosis* DNA enables sensitive detection of paucibacillary tuberculosis: a molecular diagnosis study

Contents

[Key reagents 3](#_Toc225021045)

[Verification of Salivary Nucleic Acids Derived from Exosomes 3](#_Toc225021046)

[Supplementary Experiment on the Limit of Detection of Dual-Gene qPCR Melting Curve Assay (DGPMC) 4](#_Toc225021047)

[Supplementary tables 1 Around April 2023: Optimization and Adjustment of the qPCR 5](#_Toc225021048)

[Supplementary tables 2 The ExoNA kit formulation. 6](#_Toc225021049)

[Supplementary tables 3 7](#_Toc225021050)

[Supplementary Figure 8](#_Toc225021051)

[Supplementary Figure 1 8](#_Toc225021052)

[Supplementary Figure 2 10](#_Toc225021053)

[Supplementary Figure 3 11](#_Toc225021054)

[Supplementary Figure 4 11](#_Toc225021055)

[Supplementary Figure 5 12](#_Toc225021056)

[Supplementary Figure 6 12](#_Toc225021057)

[Supplementary Figure 7 13](#_Toc225021058)

[Supplementary Figure 8 13](#_Toc225021059)

[Supplementary Figure 9 14](#_Toc225021060)

[Supplementary Figure 10 15](#_Toc225021061)

[Supplementary Figure 11 16](#_Toc225021062)

# Key reagents

DNA polymerase (Cat. No. 13689ES76) and qPCR SYBR Green Master Mix (Cat. No. 11202ES08) were purchased from YEASEN Biotechnology Co., Ltd. (Shanghai, China). TE buffer (Cat. No. B5410190100) and nuclease-free water (Cat. No. A5001970500) were obtained from Sangon Biotech Co., Ltd. (Shanghai, China). ExoNA Exosome Concentration Solution (ECS) and the DNA Extraction Kit (Cat. No. LS2101; Supplementary Table 2) were obtained from Shanghai Liquid-Bio Biotechnology Co., Ltd. The *Mycobacterium tuberculosis* H37Rv strain was obtained from the NIH. ExoQuick (Cat. No. EXOQ20a-1) was purchased from System Biosciences, LLC.

# Verification of Salivary Nucleic Acids Derived from Exosomes

In brief, 280 μL of saliva samples were initially centrifuged at 3000 g for 10 minutes. Then, the supernatant was mixed with 420 μL exosome precipitation solution (kit components 1) and incubated on ice or at 4°C for 1 hour. After incubation, the sample is centrifuged at 3000rpm for 10 minutes. A white precipitate appears in the tube, which contains exosomes (Supplementary Figure 1). Dissolve the precipitate in 50 μL of 0.1 M phosphate-buffered saline (PBS), and transport it on ice for exosome nano-flow cytometry and nanoparticle tracking analysis (NTA) (Supplementary Figure 2-6). The white precipitate was fixed in 2% glutaraldehyde for 12–48 hours, followed by post-fixation in 1% osmium tetroxide (OsO₄) for 2 hours. Subsequently, gradient dehydration was performed using 30%–100% ethanol, and the sample was embedded in epoxy resin. Following resin polymerization, the exosome-embedded epoxy resin blocks were sectioned into 50–70 nm-thick ultrathin sections using an ultramicrotome, which were then observed under a transmission electron microscope (TEM). (Supplementary Figure 7-8)

qPCR is used to detect the housekeeping gene *GAPDH* in both the polymer precipitate and residual fluid to verify that salivary DNA is derived from exosomes.

Using DNA extracted from the polymer precipitate by Component 2 of the ExoNA as a template, qPCR analysis of *GAPDH* yielded a mean Ct value of approximately 30·7. Using DNA extracted from the residual fluid after removing the Component 2 of The ExoNA as a template, qPCR detection of *GAPDH* showed no CT value. This experiment was repeated three times, with consistent results each time (Supplementary Figures 9).

This experiment indicates that after saliva sample processing with The ExoNA, exosomal nucleic acids are mainly in the precipitate, and the residual fluid after precipitation removal contains little or no exosomal nucleic acids.

This experiment indicates that after saliva sample processing with The ExoNA, exosomal nucleic acids are mainly in the precipitate, and the residual fluid after precipitation removal contains little or no exosomal nucleic acids.

# Supplementary Experiment on the Limit of Detection of Dual-Gene qPCR Melting Curve Assay (DGPMC)

Serial dilutions of the *Mycobacterium tuberculosis* (MTB) reference strain H37Rv were prepared at final concentrations of 10, 20, 40, 80,100, 200 and 1000 CFU/mL. DNase-free water was used as the negative control (NC). By serially diluting the samples, the PCR amplification of the target genes was assessed at each concentration to evaluate the detection performance of the assay across different bacterial loads. (Supplementary Figures 10)

For the establishment of the detection limit, it is necessary to conduct at least 20 repeated tests at the detection limit concentration level, and the positive detection rate should reach 95% to be more scientific. Therefore, the experimental data are supplemented.

The minimum detection limit concentration of *IS6110* gene was 10 CFU/mL, and the positive detection rate was 100% after 20 repeated experiments. Similarly, the minimum detection limit concentration of *rpoB* was verified 20 times at 40 CFU/mL, and the positive detection rate was 100%. (Supplementary Figures 11)

# Supplementary tables 1 Around April 2023: Optimization and Adjustment of the qPCR

| The optimized reaction system after April 2023 | | | The reaction system before April 2023 | | | |
| --- | --- | --- | --- | --- | --- | --- |
| Reagent | Volume（μL） | Final conc. | Reagent | Volume（μL） | Final conc. |  |
| ArtiCanATM SYBR qPCR Mix-UDG (Low ROX Premixed) | 25 | 1ⅹ | Hieff® qPCR SYBR Green Master Mix (Low Rox Plus) | 15 | 1ⅹ |  |
| *IS6110/rpoB* Forward Primer(10μM) | 1 | 0.1 μM | Uracil DNA Glycosylase(UDG),heatlabile (1 U/μL) | 0.6 | 0.02 U/μL |  |
| *IS6110/rpoB* Reverse Primer (10μM) | 1 | 0.1 μM | *IS6110/rpoB* Forward Primer(10μM) | 0.6 | 0.2 μM |  |
| *GAPDH* Forward Primer (10μM) | 0.4 | 0.2 μM | *IS6110/rpoB* Reverse Primer (10μM) | 0.6 | 0.2 μM |  |
| *GAPDH* Reverse Primer (10μM) | 0.4 | 0.2 μM |  |  |  |  |
| Sample | ≤22 | / | Sample | ≤13 | / |  |
| Add ddH_2_O to total volume | 50 | / | Add ddH_2_O to total volume | 30 |  |  |

**Note:** After optimization of the PCR system, the detection sensitivity was improved. The minimum detection limit (MDL) of the *rpoB* gene was reduced from approximately 320 CFU/mL to 40 CFU/mL, and that of the *IS6110* gene was reduced from approximately 40 CFU/mL to 10 CFU/mL.

# Supplementary tables 2 The ExoNA kit formulation.

| Component 1 | Proportion (%) | Function |
| --- | --- | --- |
| Exosome Precipitation Solution | 5 | Selectively precipitate exosomes by spatial exclusion and dehydration effects. |
| Component 2  Combination of lysis buffer and magnetic beads | Proportion (%) |  |
| LiCl | 0.5 | nucleases inhibition helper |
| Proteinase K | 1 | Degrade proteins and eliminate nuclease activity |
| Guanidine thiocyanate | 2.5 | Strong denaturants, destruction biofilms and nucleases;separate nucleic acid from protein |
| Trisodium citrate | 3 | Inhibition of metal ion-dependent nucleases; PH buffer |
| Sodium N-lauroylsarcosinate | 2 | Destruction biofilms and nucleases |
| 2-mercaptoethanol | 1 | Inhibit RNase activity; prevent nucleic acid oxidative damage |
| Isopropanol | 1 | Reduce the solubility of nucleic acids and precipitation helper |
| EDTA | 0.5 | Chelator, inhibition of metal ion-dependent nucleases |
| nuclease- free water | 90 | Provide a nuclease-free environment |

# Supplementary tables 3

| ***rpoB*** | **CT** **value** | | | | | | | |
| --- | --- | --- | --- | --- | --- | --- | --- | --- |
| **ExoNA** | 32.8 | 33.8 | 34.8 | 34.7 | 32.4 | 32.9 | 32.7 | 34.5 |
| **ExoQuick** | 32.0 | 33.3 | 32.9 | 33.8 | 31.2 | 32.7 | 32.2 | 33.4 |
| ***IS6110*** | **CT value** | | | | | | | |
| **ExoNA** | 29.0 | 25.8 | 28.9 | 27.9 | 28.5 | 27.7 | 30.8 | 29.4 |
| **ExoQuick** | 26.1 | 29.4 | 28.8 | 25.9 | 27.3 | 26.8 | 30.1 | 29.5 |

**Note:** Comparison of CT values assessed using the unpaired t test. No significant differences were observed in the CT values of *rpoB* (*P* = 0.0853) and *IS6110* (P = 0.4965); ns represents no significant difference with *P* > 0.05.

# Supplementary Figure


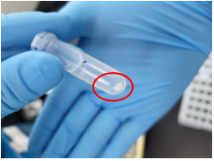


# Supplementary Figure 1

The white precipitate contains exosomes required for detection.


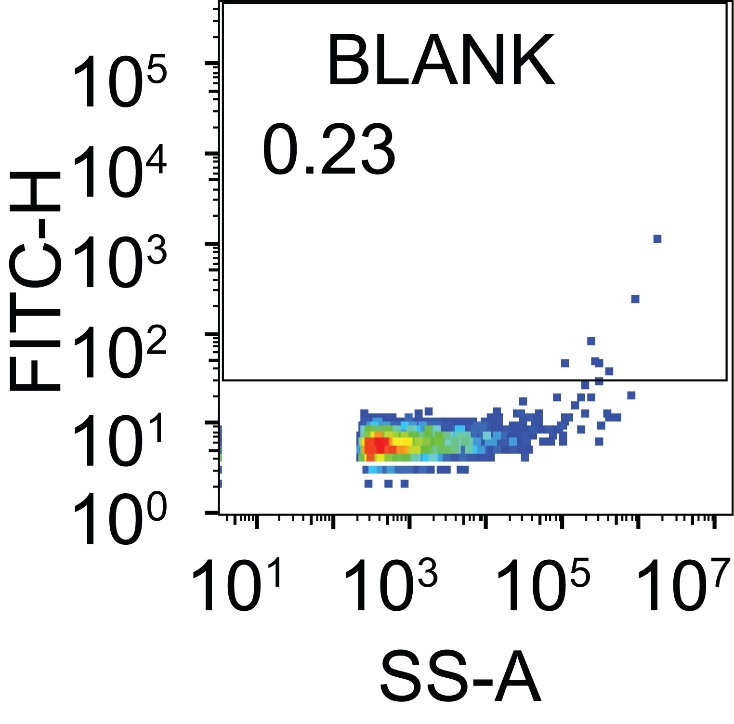


# Supplementary Figure 2

Nanoparticle flow cytometry verified the exosomal proteins CD9, CD63, and CD81.


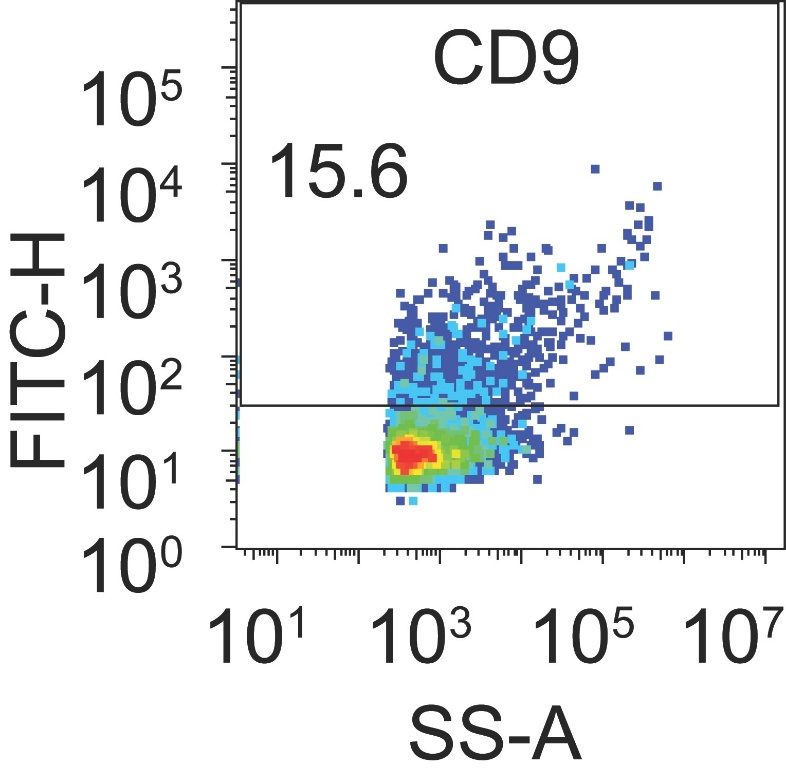


# Supplementary Figure 3

Nanoparticle flow cytometry analysis of exosomal CD9. SS-A vs FITC-H dot plot shows 15.6% positive events in the gated population.


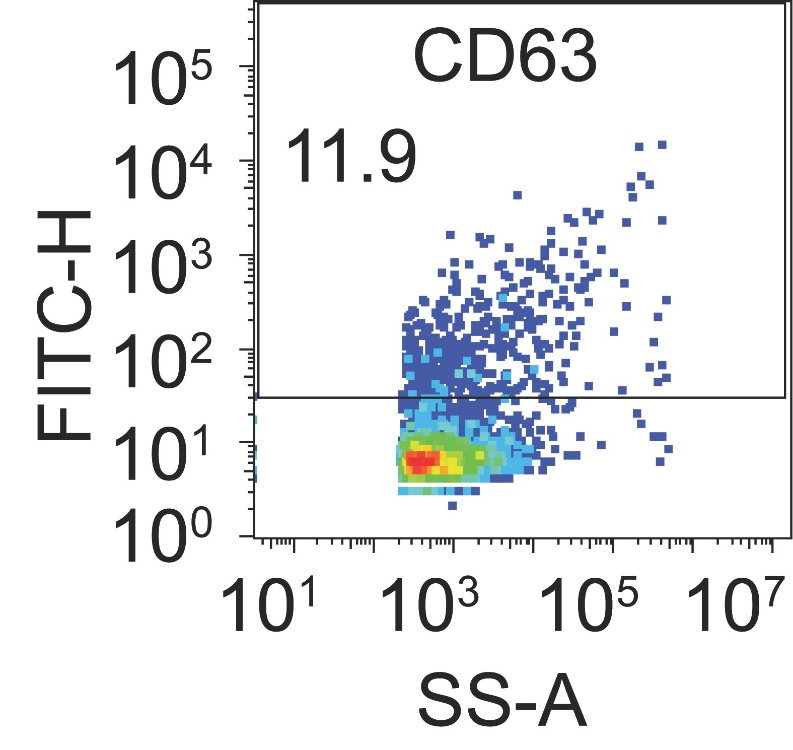


# Supplementary Figure 4

Nanoparticle flow cytometry analysis of exosomal CD63. SS-A vs FITC-H dot plot shows 11.9% positive events in the gated population.


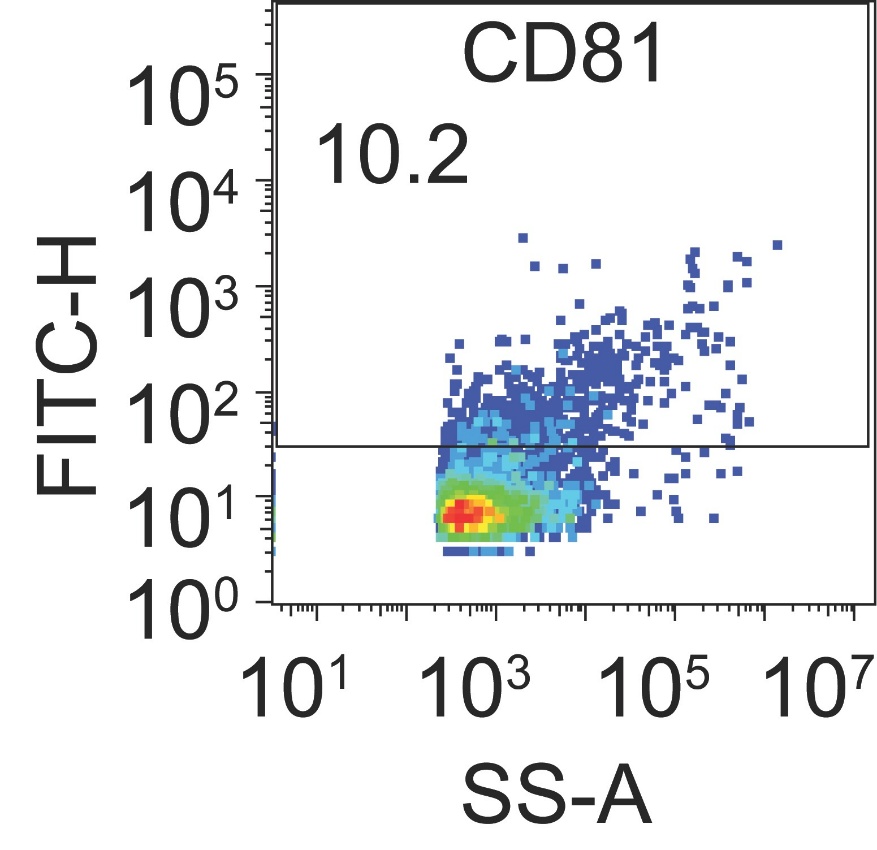


# Supplementary Figure 5

Nanoparticle flow cytometry analysis of exosomal CD81. SS-A vs FITC-H dot plot shows 10.2% positive events in the gated population.


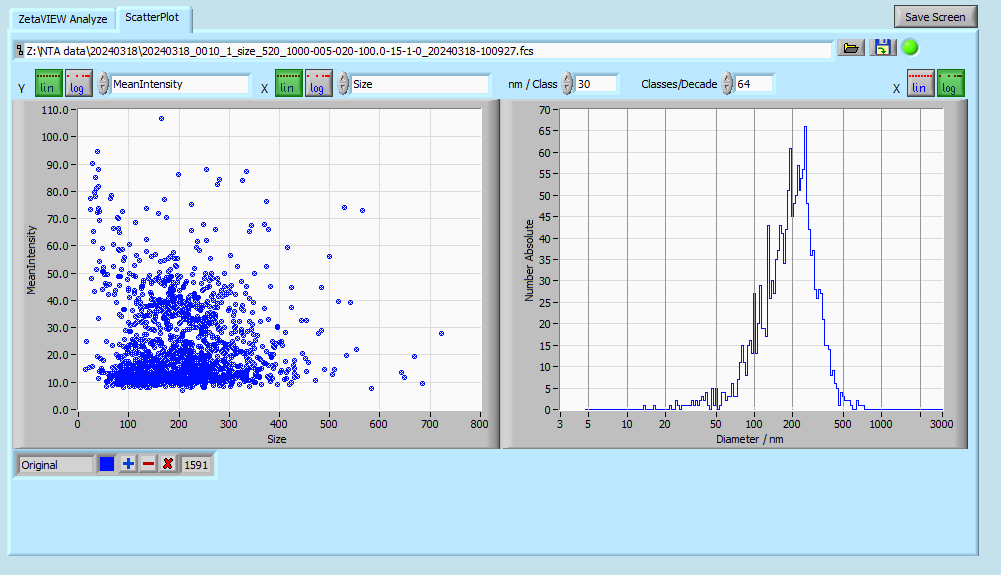


# Supplementary Figure 6

Nanoparticle tracking analysis (NTA) of isolated exosomes measured via a ZetaView system. Left: Scatter plot of mean intensity versus particle size; Right: Particle size distribution histogram, showing a peak diameter within the characteristic size range of exosomes (30–150 nm).





# Supplementary Figure 7

Representative transmission electron microscopy (TEM) image of isolated exosomes, showing characteristic cup-shaped bilayer membrane vesicles. Scale bar: 200 nm.





# Supplementary Figure 8

TEM image of exosomes with typical bilayer membrane structure. Scale bar: 100 nm.


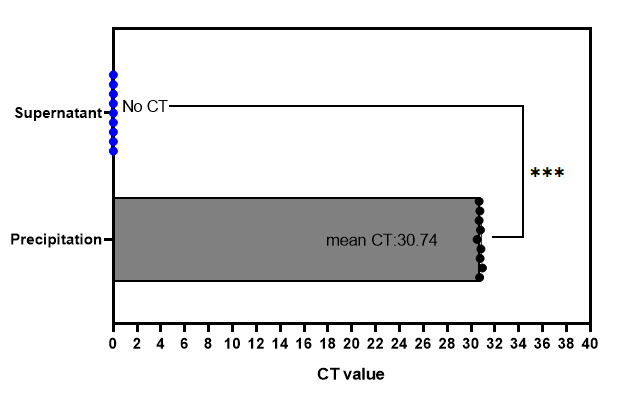


# Supplementary Figure 9

qPCR Ct values of *GAPDH* in the residual fluid (Supernatant) and polymer precipitate (extracted via ExoNA Component 2). The polymer precipitate showed a mean *GAPDH* Ct value of 30.74, while no Ct value was detected in the residual fluid. Results were consistent across 3 experimental replicates (*** indicates significant difference).


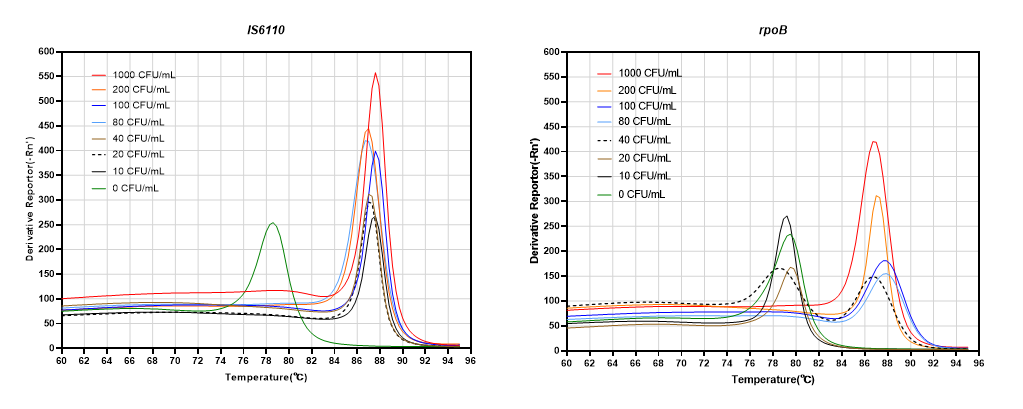


# Supplementary Figure 10

Melting curves generated from the amplification of *rpoB* and *IS6110* in *MTB* reference strain H37Rv with different concentrations. The Tm values of the specific amplicons range from 86.0°C to 88.2°C, and melting peaks below 80°C are attributed to primer dimers.

# Supplementary Figure 11

The minimum detection limit concentration of *Mycobacterium tuberculosis* *IS6110* gene was 10 CFU/mL, and the positive detection rate was 100% after 20 repeated experiments, as shown in Fig. 3a. Similarly, the minimum detection limit concentration of *Mycobacterium tuberculosis* *rpoB* was verified 20 times at 40 CFU/mL, and the positive detection rate was 100%, as shown in Fig. 3b.
